# Ⅲa期非小细胞肺癌新辅助化疗的疗效分析及应用价值

**DOI:** 10.3779/j.issn.1009-3419.2017.02.04

**Published:** 2017-02-20

**Authors:** 翠萍 唐, 思 秦, 万春 伍, 阳 吴, 涛 张

**Affiliations:** 1 400016 重庆，重庆医科大学附属第一医院肿瘤科 Depatment of Oncology, the First Affiliated Hospital of Chongqing Medical University, Chongqing 400016, China; 2 400016 重庆，重庆医科大学附属第一医院心血管内科 Department of Cardiovascular, the First Affiliated Hospital of Chongqing Medical University, Chongqing 400016, China

**Keywords:** 肺肿瘤, 新辅助化疗, 无病生存期, 疗效分析, Lung neoplasms, Neoadjuvant chemotherapy, Disease-free survival, Efficacy

## Abstract

**背景与目的:**

新辅助化疗应用于可手术切除的Ⅲa期非小细胞肺癌（non-small cell lung cancer, NSCLC）患者的确切疗效及安全性尚存争议。本研究旨在探讨新辅助化疗对可手术切除Ⅲa期NSCLC患者的近期疗效，并分析其与术后并发症的相关性。

**方法:**

根据纳入及排除标准，回顾性分析2011年1月-2013年10月重庆医科大学附属第一医院收治的明确临床诊断为Ⅲa期NSCLC 370例患者完整资料，根据术前是否接受新辅助化疗分为两组，其中A组为新辅助化疗+手术组97例，B组为直接手术组273例，比较两组患者的临床资料，分析新辅助化疗后肿瘤降期率，并将两组患者的手术情况、术后并发症进行对比，统计两组患者3年无病生存期（disease-free survival, DFS）。

**结果:**

A组患者新辅助化疗后肿瘤总降期率为65.98%（64/97）；两组患者R0切除率分别为96.91%（94/97）和90.48%（247/273），手术时间、术中出血量、术后平均住院日差异均无统计学意义（*P* > 0.05）；术后并发症总发生率A组稍高于B组，分别为76.29%（74/97）和72.52%（198/273），差异无统计学意义（*P* > 0.05）；所有患者术后随访2个月-36个月，中位随访时间12.7个月，两组患者术后总体复发转移率分别为63.92%（62/97）和94.87%（259/273），有统计学差异（*P* < 0.05）；A、B两组患者中位DFS分别为19.46个月和11.34个月，差异有统计学意义（*P* < 0.001）。

**结论:**

新辅助化疗可使Ⅲa期NSCLC患者受益，能有效降低肿瘤分期，提高肿瘤切除率，可降低术后局部复发率及远处转移率，提高患者的无进展生存期；且并不明显增加术后并发症的发生率。

## Abstract (en)

**Background and objective:**

The therapeutic effect and side effect of neoadjuvant chemotherapy were still disputing issues when applied to resectable Ⅲa stage non-small cell lung cancer (NSCLC) patients. The retrospective analysis was aimed to investigate the short-term efficacy and postoperative complications in resectable Ⅲa NSCLC patients treated with neoadjuvant chemotherapy.

**Methods:**

According to inclusion criteria and exclusion criteria, 370 patients with clinical diagnosis of Ⅲa NSCLC were selected from our hospital between January 2011 and October 2013 were retrospectively analyzed. According to treatment method, group A (preoperative neoadjuvant chemotherapy+surgery group) contained 97 cases, and 273 patients were included in group B (direct surgery without neoadjuvant treatment group). The clinical data, surgical approach, main postoperative complications and disease-free survival (DFS) among patients in two groups were recorded.

**Results:**

The total down-staging in group A was 65.98% (64/97), the R0 resection in group A and group B were 96.91% (94/97) and 90.48% (247/273), respectively. The operation time, bleeding, postoperative hospitalization were no statistical difference (*P* > 0.05), and the main postoperative complications of the patients in two groups were 76.29% (74/97) and 72.52% (198/273) (*P* > 0.05). All patients followed-up for 2 months-36 months, the median follow-up time was 12.7 months, the total recurrence and metastasis rates were 63.92% (62/97) and 94.87% (259/273) (*P* < 0.05) and the median DFS were 19.46 months and 11.34 months (*P* < 0.001).

**Conclusion:**

Neoadjuvant chemotherapy can benefit patients of Ⅲa stage NSCLC partly in downstaging T and N stage in tumor, enhance the R0 resection, but not significantly increased the postoperative complications of the patients, which can reduce the local recurrence and metastasis, enhance the DFS effectively.

原发性肺癌是全世界范围内最常见的恶性肿瘤^[1]^，我国是肺癌的高发国家，每年患病率约为130.2（1/10万）^[2]^，其中非小细胞肺癌（non-small cell lung cancer, NSCLC）约占75%-80%^[3]^。手术为治疗NSCLC的主要手段，然而大部分患者在就诊时就已是局部晚期或已发生转移，单纯手术很难达根治性切除，整体疗效及预后较差。

近年来，随着肿瘤综合治疗研究的进展，新辅助化疗的概念被提出，大量针对Ⅲa期NSCLC新辅助化疗相关临床研究涌现，在多项前瞻性随机对照研究中，含铂类的双药联合新辅助化疗方案在NSCLC患者中显示出良好的应用前景，可提高包括Ⅲ期在内的NSCLC患者5年生存率约5%^[4]^，但目前对于新辅助化疗应用于可手术切除的Ⅲa期NSCLC患者的确切疗效尚存争议，且其有效性及安全性有待进一步验证。本研究旨在通过对比Ⅲa期NSCLC患者新辅助化疗后肿瘤-淋巴结-转移（tumor-nodemetastasis, TNM）分期变化，以及与单纯手术组相比其R0切除率、手术并发症、局部复发及远处转移等相关因素差异性，并对比两组患者3年无病生存期（disease-free survival, DFS），探讨新辅助化疗在可手术切除Ⅲa期NSCLC中的术后并发症及近期疗效，为临床上Ⅲa期NSCLC患者的治疗方案选择提供依据。

## 1 资料与方法

### 1.1 一般资料

#### 1.1.1 纳入标准与排除标准

纳入标准：①经纤维支气管镜、经皮穿刺肺组织活检、纵隔淋巴结活检或超声内镜（endosonographic procedures, EBUS/BUS）^[5]^等证实为NSCLC初治患者；②治疗前经胸部增强计算机断层扫描（computed tomography, CT）或正电子发射型计算机断层显像（positron emission computed tomography, PET）-CT、头颅磁共振成像（magnetic resonance imaging, MRI）、全身骨扫描、腹部CT检查除外脑、骨、肝等转移，并证实肿瘤临床分期为Ⅲa期（T1-2N2M0、T3N1-2M0、T4N0-1M0）；③术后均应行4周期辅助化疗；④既往无恶性肿瘤病史。排除标准：①无明确组织学或细胞学诊断；②接受过化疗或其他相关抗肿瘤治疗；③有严重心脏、肝脏或肾脏疾病，不能耐受手术或预期生存时间不长者；④术后未完成化疗或于外院行化疗；⑤新辅助化疗期间因副反应终止原方案者；⑥随访期间失访或非疾病相关死亡者。

#### 1.1.2 研究对象

根据纳入及排除标准，共收集重庆医科大学附属第一医院2011年1月-2013年10月收治的明确诊断为Ⅲa期NSCLC 370例患者完整资料，根据术前是否接受新辅助化疗分为两组，其中A组为新辅助化疗+手术组共97例，B组为直接手术组共273例。A组97例患者中，男性75例（77.32%），女性22例（22.68%），中位年龄57.3（36-73）岁；B组273例患者中，男性207例（75.82%），女性66例（24.18%），中位年龄57.7（20-78）岁。一般资料见表 1。两组患者在年龄组成、性别、肿瘤病理类型、肿瘤分期等方面比较差异均无统计学意义（*P* > 0.05）。

**1:** 两组患者一般临床资料[*n*%)] Clinical characteristics of patients in two groups [*n* (%)]

Characteristics	Group A (*n*=97)	Group B (*n*=273)
Gender		
Male	75 (77.32%)	207 (75.82%)
Female	22 (22.68%)	66 (24.18%)
Age (yr)		
≤55	33 (34.02%)	100 (36.63%)
> 55	64 (65.98%)	173 (63.37%)
Smoking history		
Yes	72 (74.23%)	186 (68.13%)
No	25 (25.77%)	87 (31.87%)
Histology		
Squamous	57 (58.76%)	118 (43.22%)
Adenocarcinoma	37 (38.15%)	136 (49.82%)
Adenosquamous	3 (3.09%)	19 (6.96%)
Location		
Central	55 (56.70%)	145 (53.11%)
Peripheral	42 (43.30%)	128 (46.89%)
TNM stage		
T1-2N2M0	51 (52.58%)	161 (58.97%)
T3N1-2M0	46 (47.42%)	85 (31.14%)
T4N0-1M0	0 (0.00%)	27 (9.89%)
TNM: tumor-node-metastasis. Group A: preoperative neoadjuvant chemotherapy+surgery group; Group B: direct surgery without neoadjuvant treatment group.		

### 1.2 方法

#### 1.2.1 新辅助化疗方案

根据美国国立综合癌症网络（National Comprehensive Cancer Network, NCCN）指南，A组患者术前选择紫杉醇、吉西他滨、培美曲塞或多西他赛联合铂类的化疗方案^[6]^，根据患者一般情况，选择2个-3个周期疗程。

#### 1.2.2 手术治疗

A组患者化疗结束后4周-5周行手术，B组患者入院后直接手术，术前完善心电图、胸部+腹部增强CT、头颅MRI、全身骨扫描、血常规、生化常规、凝血功能、肿瘤标志物等辅助检查，全面评估患者一般情况及肿瘤负荷，排除手术禁忌后选择恰当的手术方式，主要包括楔形切除、肺叶切除、袖式肺叶切除、全肺切除等方式。

#### 1.2.3 术后辅助治疗

根据患者一般情况，所有患者均于术后14天-28天起予以辅助化疗，根据NCCN指南建议，术后一般选用GP（Gemcitabine+platinum）、TP（paclitaxe+platinum）、PP（pemetrexed+platinum）、DP（docetaxel+platinum）等化疗方案行4个周期辅助化疗，对N2及手术切缘阳性患者同时辅以术后放疗。

### 1.3 新辅助化疗疗效判断

根据影像学检查结果，按照国际肺癌协会（International Association for the Study of Lung Cancer, IASLC）2009年颁发的UICC第7版肺癌TNM分期标准，Ⅲa期包括：T1-2N2M0、T3N1-2M0、T4N0-1M0，比较A组患者新辅助化疗前后肿瘤分期变化，对比A组和B组患者肿瘤达R0切除比例、手术时间、手术并发症（术中出血，术后肺部感染、胸腔积液、胸腔积气等）、术后平均住院日及3年DFS（局部复发和远处转移）等指标是否有差异。

### 1.4 随访

第1年按照每3个月随访1次，之后每6个月随访1次，2年后每年复查1次，直至术后3年，复查内容包括胸部+腹部增强CT、头颅MRI、全身骨扫描等指标。DFS时间计算从手术日期开始。随访方式采用电话随访。

### 1.5 统计学分析

应用SPSS 19.0统计软件进行数据统计学分析，组间计量数据比较采用*t*检验，计数资料的比较采用*χ*^2^检验，采用*Kaplan*-*Meier*方法进行无病生存期分析，*Log*-*rank*检验进行差异性分析，*P* < 0.05为差异有统计学意义。

## 2 结果

### 2.1 新辅助化疗前后肿瘤分期的比较

A组患者新辅助化疗方案选择：GP方案43.30%（42/97），DP方案27.83%（27/97），TP方案22.68%（22/97），PP方案6.19%（6/97）。新辅助化疗完成后行胸部+腹部增强CT、头颅MRI、全身骨扫描等全面检查，其结果提示所有患者均可行手术切除，术后病理分期与新辅助化疗前比较：T分期中，共52例（53.60%）分期下降，其中T3降为T2/T1者共34例（35.05%），T2降为T1者18例（18.56%）；N分期中，共63例（64.95%）分期下降，其中N2降为N1/N0共53例（54.64%），N1降为N0者10例（10.31%）。结果见表 2。新辅助化疗后总降期率达65.98%（64/97）。

**2:** 两组患者手术情况及术后病理情况比较[*n* (%)] The comparation of surgical and postoperative pathological staging in two groups [*n* (%)]

Item	Group A (*n*=97)	Group B (*n*=273)	*P* value
Surgical resection			
Wedge resection	8 (8.25%)	7 (2.56%)	
Lobectomy	79 (81.45%)	215 (78.76%)	
Sleeve resection	6 (6.18%)	21 (7.69%)	
Pneumonetomy	4 (4.12%)	30 (10.99%)	0.044, 4
Incisal margin			0.042, 9
R0	94 (96.91%)	247 (90.48%)	
R1	3 (3.09%)	26 (9.52%)	
c-T			
T1	6 (6.19%)		
T2	45 (46.39%)		
T3	46 (47.42%)		
T4	0 (0.00%)		
c-N			
N0	0 (0.00%)		
N1	13 (13.40%)		
N2	84 (86.60%)		
p-T			
T1	29 (29.90%)	22 (8.06%)	
T2	56 (57.73%)	139 (50.92%)	
T3	12 (12.37%)	85 (31.13%)	
T4	0 (0.00%)	27 (9.89%)	
pT down-staging	52 (53.60%)		
T3→T2/T1	34 (35.05%)		
T2→T1	18 (18.56%)		
p-N			
N0	43 (44.33%)	24 (8.79%)	
N1	23 (23.71%)	33 (12.09%)	
N2	31 (31.96%)	216 (79.12%)	
pN down-staging	63 (64.95%)		
N2→N1/N0	53 (54..64%)		
N1→N0	10 (10.31%)		

### 2.2 两组患者手术情况比较

所有患者均行手术治疗，手术情况见表 2，其中A组单侧全肺切除4例（4.12%），R0切除率96.91%（94/97）；B组患者共30例（10.99%）行单侧全肺切除，R0切除率90.48%（247/273）。新辅助化疗降低了全肺切除的比例，提高了R0切除率，而两组患者手术时间、术中出血量、术后平均住院日相比，均无统计学差异（*P* > 0.05）。见表 2、表 3。

**3:** 两组患者手术情况比较（Mean±SD） The general situation of operation in two groups (Mean±SD)

	Group A	Group B	*P* value
Operation time (min)	203.09±54.73	212.67±62.24	0.348, 8
Bleeding (mL)	260.17±188.61	282.35±379.53	0.664, 2
Postoperative hospitalization (d)	9.82±3.96	10.05±4.19	0.719, 5

### 2.3 术后并发症

两组患者术后主要并发症发生率（术后感染、胸腔积液、胸腔积气等）等方面差异无统计学意义（*P* > 0.05），总并发症发生率分别为76.29%（74/97）和72.52%（198/273），无统计学差异（*P* > 0.05）。结果见表 4。

**4:** 两组患者术后主要并发症比较[*n* (%)] The main postoperative complications of the patients in two groups [*n* (%)]

	Group A (*n*=97)	Group B (*n*=273)	*P* value
Postoperative infection	50 (51.55%)	138 (50.55%)	0.866, 0
Pleural effusion	42 (43.30%)	127 (46.52%)	0.584, 3
Aerothorax	30 (30.93%)	60 (21.98%)	0.077, 6
Total incidence of complications	74 (76.29%)	198 (72.52%)	0.746, 2

### 2.4 术后辅助放化疗

两组患者术后辅助放化疗情况见表 5，A组患者术后放疗患者共35例，辅助化疗方案选择：TP方案25.77%（25/97），GP方案39.18%（38/97），DP方案29.90%（29/97），PP方案5.15%（5/97）；B组患者术后放疗共242例，辅助化疗方案选择：TP方案31.13%（85/273），GP方案36.26%（99/273），DP方案28.21%（77/273），PP方案4.40%（12/273）。

**5:** 两组患者术后辅助放化疗情况[*n* (%)] The adjuvant radiotherapy and chemotherapy in two groups [*n* (%)]

Characteristics	Group A (*n*=97)	Group B (*n*=273)
Chemotherapy regimens		
Paclitaxe+platinum (TP)	25 (25.77%)	85 (31.13%)
Gemcitabine+platinum (GP)	38 (39.18%)	99 (36.26%)
Docetaxel+platinum (DP)	29 (29.90%)	77 (28.21%)
Pemetrexed+platinum (PP)	5 (5.15%)	12 (4.40%)
Adjuvant radiotherapy		
Yes	35 (36.08%)	242 (88.64%)
No	62 (63.92%)	31 (11.36%)

### 2.5 随访

患者术后定期复查，包括病史、体检、肿瘤标志物、影像学检查（胸部增强CT、腹部彩超、头颅增强MRI及全身骨扫描）等^[7]^，所有病例随访时间为2个月-36个月，截止时间2016年10月，中位随访时间12.7个月，A组3年内复发转移（包括局部复发、全身远地转移、复发转移均有者）率63.92%（62/97），3年中位DFS 19.46个月；B组3年内复发转移率94.87%（259/273），3年中位DFS 11.34个月；B组3年复发转移率显著高于A组，差异有统计学意义（*P* < 0.05）。两组患者无DFS结果见图 1，差异有统计学意义（*P* < 0.001）。新辅助化疗在控制复发转移率及延长患者DFS方面体现出一定的优势。

**1:**
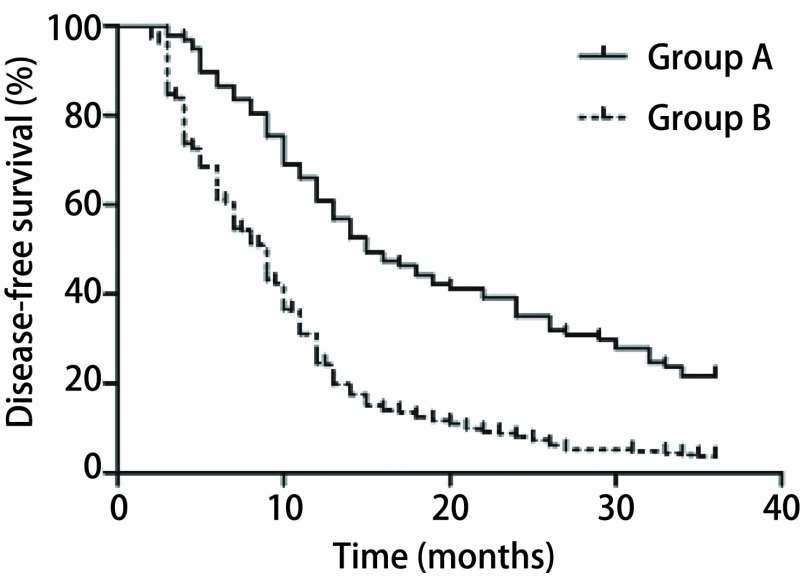
两组NSCLC患者的无病生存曲线 Disease-free survival curve of the NSCLC patients in two groups

## 3 讨论

NSCLC是一种全身性疾病，既有局部病灶，又有全身多处微转移，对于可手术切除患者，术后辅助化疗或术前新辅助化疗用于提高NSCLC患者无进展生存期（progression-free survival, PFS）和总生存期（overall survival, OS）^[3]^。2016年NCCN指南最新建议：Ⅲa期患者术后应常规行化疗或联合放疗。然而对于术前新辅助化疗能否使Ⅲa期NSCLC患者获益，目前尚未定论^[8]^。

新辅助化疗是指在手术前进行的化疗，可通过控制及缩小原发病灶、消除微小转移灶等方式减少肿瘤负荷及降低术后复发率，并一定程度提高肿瘤患者远期生存率，较直接手术能使肿瘤患者获益。2013年Horita^[9]^的*meta*分析纳入了7项临床研究共1, 447例患者，其结果提示：对于可手术切除的Ⅲa期NSCLC患者，术前新辅助化疗较术后辅助化疗在远期生存方面稍显优势，相对风险比更低（0.77 *vs* 0.83）。尽管许多医生已将新辅助化疗运用于Ⅲa期NSCLC的治疗中，然而随着研究的不断深入，质疑之声不断出现，一些学者认为，新辅助化疗可能致部分患者因原发耐药丧失根治性手术机会，或增加早期术后复发率。Berghmans^[10]^对1993年-2003年发表的6个Ⅲ期前瞻性临床研究进行*meta*分析后认为，对于Ⅲa期NSCLC患者，新辅助化疗显示出有益于生存的趋势，但其结果无统计学差异。Nakamura^[11]^分析了376例Ⅲa期患者的资料，认为尽管新辅助化疗对于Ⅲa期NSCLC患者显示出有益于1年、3年、5年OS的趋势，但其与直接手术者相比差异无统计学意义（*P*=0.052、0.108和0.212）。然而，本研究结果显示，新辅助化疗能明显提高患者3年DFS（19.46个月*vs* 11.34个月，*P* < 0.001），这与前面*meta*分析结果不一致，可能与*meta*分析中纳入的临床研究时间跨度太大，所使用化疗方案与现在指南推荐方案疗效存在差异，以及既往研究中临床分期评估手段的局限性等有关，故各临床研究结果虽提示新辅助化疗对可手术切除的Ⅲa期NSCLC患者与直接手术相比有优势，但经*meta*分析却无统计学差异。

本研究回顾性分析了本院370例临床分期为Ⅲa期患者，根据术前是否接受过新辅助化疗分为两组，通过对随访资料的回顾性分析，结果提示：新辅助化疗可以使敏感的肿瘤组织明显缩小，肿瘤总病理降期率达65.98%（64/97）；与直接手术相比，新辅助化疗能降低全肺切除率（4.12% *vs* 10.99%, *P* < 0.05），最大程度保留正常组织，提高患者术后生活质量。新辅助化疗组与直接手术组相比，肿瘤R0切除率明显提高，差异有统计学意义（96.91% *vs* 90.48%, *P* < 0.05）。新辅助化疗可降低肿瘤浸润深度及肿瘤细胞活性、侵袭性，即使术中脱落或外周血循环中残存肿瘤细胞术后也难以生长、繁殖，从而降低了肿瘤术后局部复发和远处转移发生率。相关研究表明，新辅助治疗后病理性降期能明显改善患者的预后^[12, 13]^。本研究结果显示，新辅助化疗组总复发转移率（63.92% *vs* 94.87%, *P* < 0.05）及DFS（19.46个月*vs* 11.34个月，*P* < 0.001）均明显优于直接手术组。然而Toyokawa等^[14]^进行的一项Ⅲ期临床试验却指出，病理反应及纵隔降期并未给患者带来更好的生存获益，可能与该试验中完整切除率较低而全肺切除率较高有关。一些研究者提出，针对可切除的Ⅲa期患者，新辅助化疗可能因患者对化疗不敏感导致病情进展而错过最佳手术时机。本研究中，新辅助化疗后患者手术完成率为100%，术后并发症A组稍高于B组，但并无统计学差异（76.29% *vs* 72.52%, *P* > 0.05），证实了术前新辅助化疗的可行性。

Ⅲa期NSCLC具有异质性，纵隔淋巴及受累情况、淋巴及大小、有无融合均与患者预后密切相关^[15]^，单纯外科手术对于局部晚期Ⅲa期NSCLC患者疗效差。本研究结果表明：新辅助化疗能有效降低肿瘤分期，提高肿瘤切除率，降低术后局部复发率及远处转移率，提高患者DFS，并不明显增加术后并发症的发生率。提示新辅助化疗结合手术治疗可使部分Ⅲa期NSCLC患者受益，这与先前的一些研究结论相符合^[16, 17]^，可推荐用于部分Ⅲa期NSCLC可手术切除患者。但本研究为单中心回顾性分析，存在一定局限性及选择偏倚，更多结论需大量前瞻性研究分析。
